# Successful laparoscopic surgery combined with selective arterial embolization for bleeding due to jejunal angiodysplasia: a case report

**DOI:** 10.1186/s12893-020-00924-3

**Published:** 2020-10-31

**Authors:** Hitoshi Hara, Soji Ozawa, Kazuhito Nabeshima, Jun Koizumi

**Affiliations:** 1 Department of Gastroenterological Surgery, Tokai University School of Medicine, Isehara, Kanagawa Japan; 2 Department of Radiology, Tokai University School of Medicine, Isehara, Kanagawa Japan

**Keywords:** Small intestinal angiodysplasia, Small bowel bleeding, Selective arterial embolization, Laparoscopic surgery, Indigo carmine

## Abstract

**Background:**

Angiodysplasia of the gastrointestinal tract is a rare vascular pathology that sometimes causes massive hemorrhage. Angiodysplasias are particularly difficult to find in the small intestine for anatomical reasons, often impeding their diagnosis and treatment. Lesion localization is a major challenge in cases of small bowel bleeding requiring surgical intervention.

**Case presentation:**

The present case was a 52-year-old woman who was urgently hospitalized with repeated tarry stools. Surgical intervention was chosen after conservative treatment failed to improve her condition. The source of bleeding was suspected to be a vascular lesion discovered in the small intestine during a past double-balloon endoscopy. Abdominal contrast computed tomography revealed a jejunal hemorrhage. We chose selective arterial embolization to stabilize her hemodynamics followed by surgical intervention as her treatment plan. Several embolic and contrast agents (cyanoacrylate, indigo carmine, and Lipiodol) were combined to help identify the location of the lesion during surgery. This multi-pronged approach allowed us to localize the lesion under laparoscopic guidance with high confidence and accuracy, and to excise a 6-cm segment of the small intestine. The lesion was histologically diagnosed as angiodysplasia. No re-bleeding has been observed since the operation.

**Conclusion:**

We report our experience with a case of jejunal angiodysplasia, which was localized with selective arterial embolization using an array of embolic and contrast agents, and then excised laparoscopically. Selective arterial embolization with indigo carmine dye to treat small bowel bleeding preoperatively not only makes the surgery safer by stabilizing the patient’s hemodynamics, but is also very useful for localizing the lesion intraoperatively.

## Background

Angiodysplasia (AGD) of the gastrointestinal tract is a rare vascular pathology that may cause massive hemorrhage. AGDs cannot always be identified due to their small size, their confinement between the mucosal surface and the lamina propria, and their tendency to bleed intermittently. AGDs of the small intestine are particularly difficult to identify for anatomical reasons, often impeding their diagnosis and treatment [1–3]. When double-balloon enteroscopy (EBD) can identify a bleeding AGD, endoscopic hemostasis can be possible [2]. However, surgical intervention remains the most reliable treatment since rebleeding after endoscopic hemostasis is not rare [2, 4].

Lesion localization is a major challenge during surgery for AGDs and other sources of small bowel bleeding [1, 2]. This case report details our experience with one patient with gastrointestinal bleeding due to a jejunal AGD. Selective arterial embolization with a combination of embolic and contrast agents was first performed to localize the bleeding source and stabilize the patient’s hemodynamics. During subsequent laparoscopic surgery, the small intestine was partially resected to remove the affected tissue after accurately identifying the culprit lesion.

## Case presentation

**Fig. 1:**
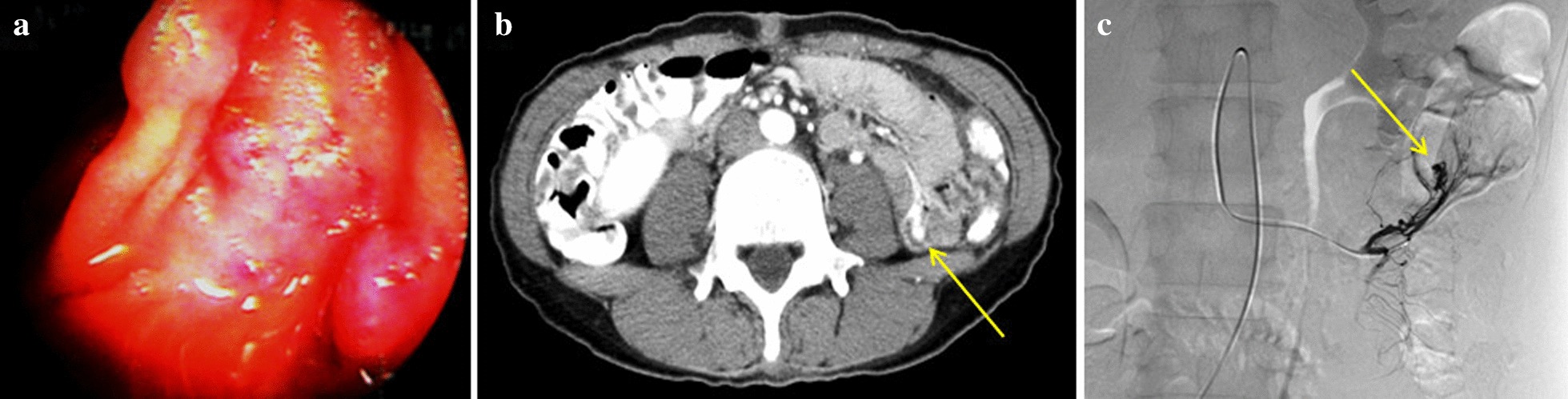
**a** Double-balloon endoscopy image. A vascular lesion is visible. **b** Contrast CT image. Arrow indicates contrast extravasation at the small intestinal wall. **c** Selective angiography images. Arrow indicates contrast extravasation peripheral to the first jejunal branch of the superior mesenteric artery

The present case was a 52-year-old woman. Her chief complaint was tarry stools. She had a history of tuberculosis diagnosed at 30 years of age. The patient had been hospitalized 8 months earlier at the same hospital’s gastroenterology department for tarry stools. During her earlier hospitalization, her hemoglobin level dropped to 5.7 g/dL, necessitating a blood transfusion. The source of the bleeding could not be identified by upper and lower gastrointestinal endoscopy. She was discharged after the tarry stools spontaneously disappeared. Later, she underwent both antegrade and retrograde DBE. A vascular lesion in the small intestine was identified during the anterograde DBE (Fig. 1a). No active bleeding was observed, but the site was still cauterized and marked with an ink tattoo.

Hemorrhagic shock due to tarry stool may have been the reason for her urgent admission to the department of gastroenterology in the present account. On admission, her blood pressure was 80/50 mmHg. Laboratory examination showed anemia with a hemoglobin level of 8.0 g/dL. After admission, conservative treatment by fasting and fluid rehydration was initially chosen, but the patient’s tarry stool persisted and her anemia worsened. Neither upper nor lower gastrointestinal endoscopy nor DBE could identify the source of the bleeding. Abdominal contrast computed tomography revealed extravasation in the small intestine in the arterial phase (Fig. 1b). The gastroenterologists suspected her bleeding to be attributable to the vascular lesion in the small intestine, which was identified in her previous DBE, and referred the patient to our department for surgery.

First, we performed abdominal angiography to identify the bleeding site. Contrast extravasation was observed peripheral to the first jejunal branch of the superior mesenteric artery. We decided to perform selective arterial embolization based on this finding. We assumed that the lesion site would be tattooed with black ink if it were the same one marked during her DBE procedures eight months earlier. However, other locations were marked with ink during the procedure, such as the deepest points reached during each approach. We were concerned that this would impede our ability to accurately choose the marking that corresponded to the lesion during the operation. Selective arterial embolization using n-butyl cyanoacrylate (Histoacryl; B.BRAUN, Melsungen, Germany) mixed with lipiodol (Guerbet, Aulnay-sous-Bois, France) was performed following the arterial injection of indigo carmine to localize the lesion during the subsequent operation. We performed surgery with the catheter still in place (Fig. 1c).

**Fig. 2:**
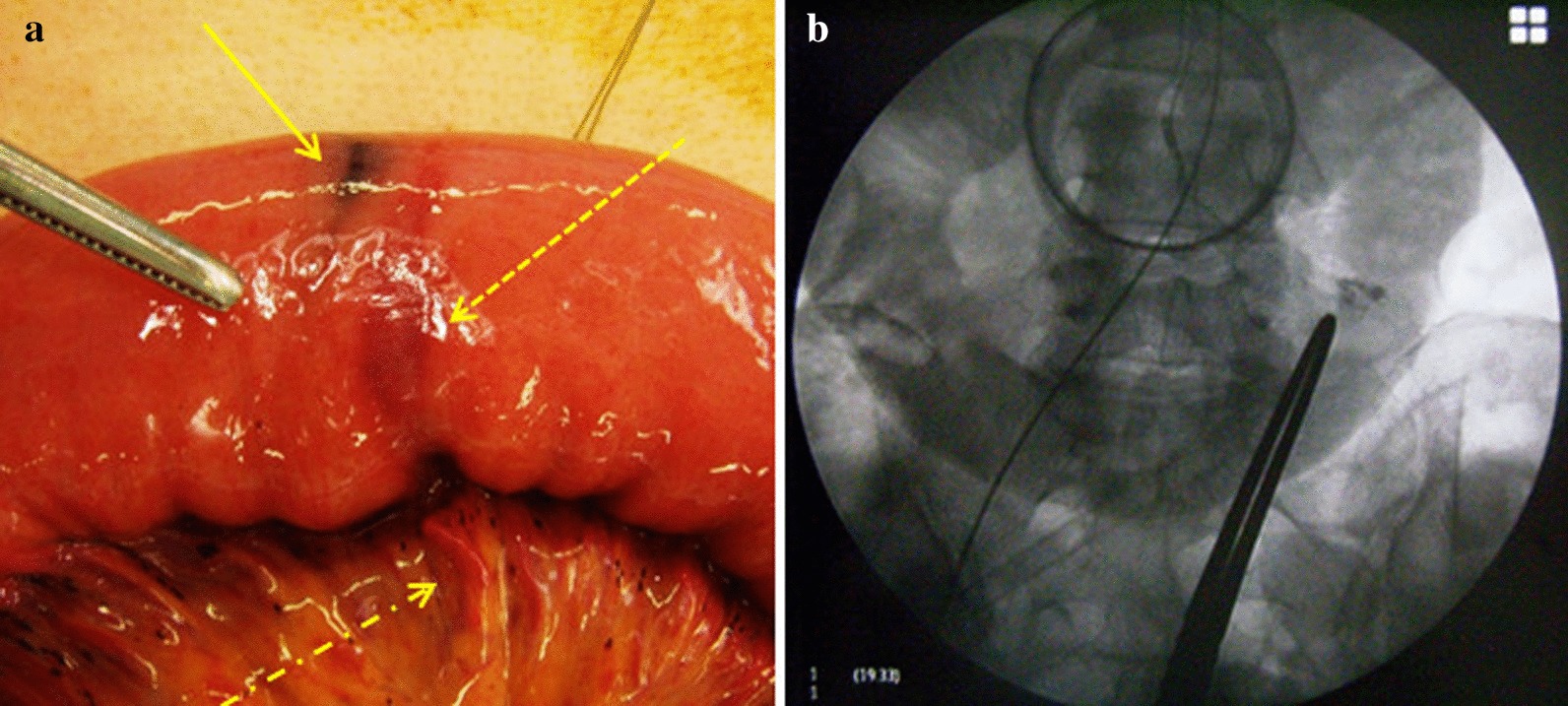
**a** Intraoperative photograph. The solid arrow indicates the ink tattoo matching the lesion site; the dashed arrow indicates the indigo carmine’s light-blue staining; the dot-dash arrow indicates the cyanoacrylate mass detected by palpation. **b** Intraoperative fluoroscopic image. Lipiodol accumulation was visualized at the same site using fluoroscopy

Laparoscopic surgery was chosen because the patient’s hemodynamics were stable following arterial embolization. To search inside the abdominal cavity, a 12-mm camera port was inserted into the umbilicus and a 5-mm port through the center of the lower abdomen. Several ink tattoos were observed in the small intestine. The umbilical port site was extended to 4 cm and fitted with a LAPDISC-mini (Hakko Co., Ltd., Nagano, Japan). We passed the small intestine through the LAPDISC-mini, which had a diameter of 70 mm, to search for the lesion extracoporeally. One ink tattoo was observed in the jejunum, approximately 30 cm distal to the ligament of Treitz. The tip of the inserted catheter and an embolized mass due to n-butyl cyanoacrylate were confirmed in the adjacent mesentery by palpation. At the same site, light-blue staining due to indigo carmine was visually confirmed in the intestinal wall, and a lipiodol embolus was observed under fluoroscopic guidance. The lesion was diagnosed as a vascular lesion and the source of the bleeding. Minimal small bowel resection could be performed to remove approximately 6 cm of the small intestine surrounding the lesion. The extracoporeal anastomosis was performed by functional end-to-end anastomosis using linear staplers. The operation time was 97 min, and the blood loss was 32 mL (Fig. 2a, b).

**Fig. 3:**
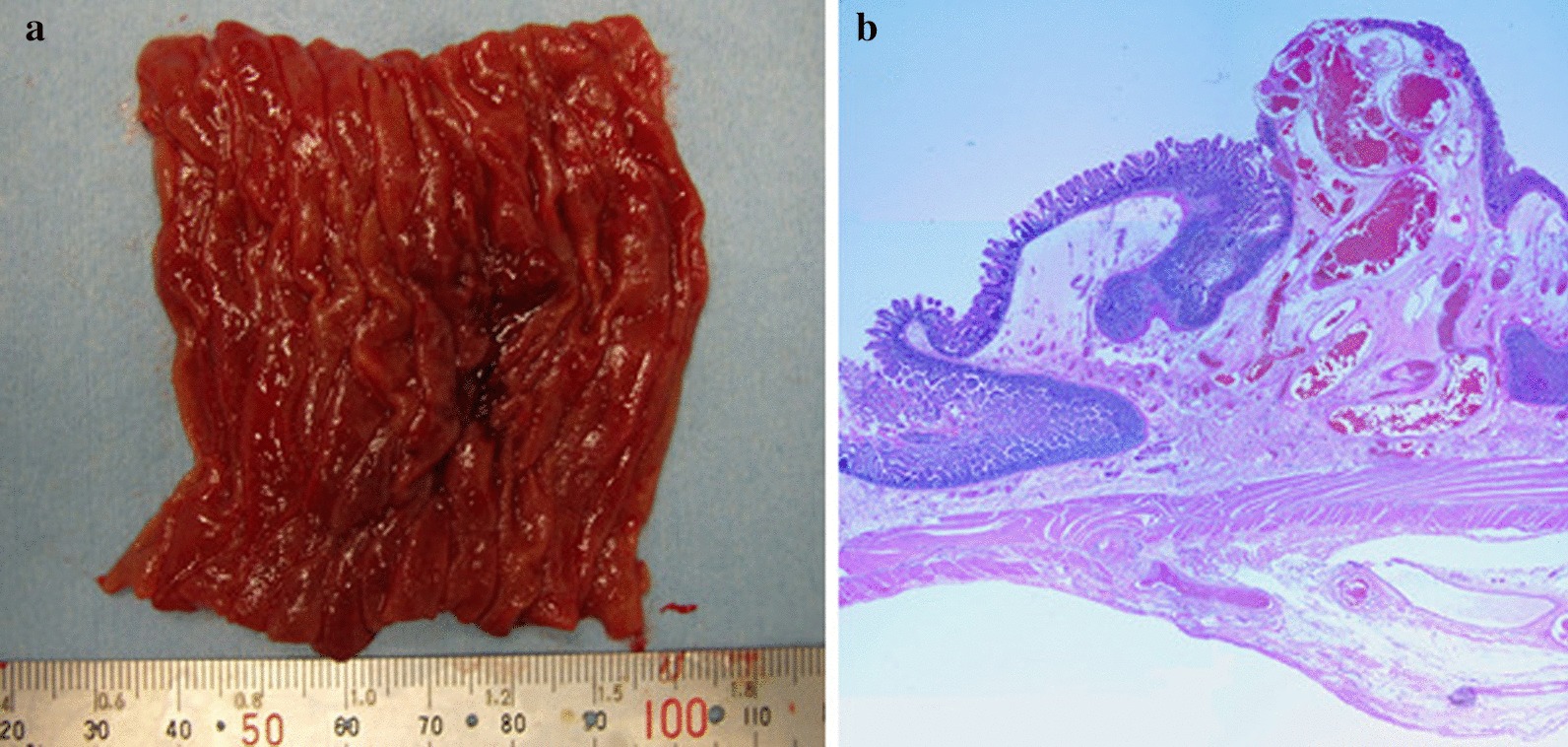
**a** Excised tissue. A polypoid lesion with a dark-red center is visible. **b** Histopathological findings. Irregularly dilated blood vessels lined by the endothelium were noted in the submucosal area. There were very few smooth muscles in the vessel wall. The submucosa was edematous, although the inflammatory findings were minor. The mucosal surface was locally eroded (Hematoxylin and Eosin staining, × 2.5)

The excised tissue showed a polypoid lesion (7 × 5 mm) with a dark-red center (Fig. 3a). Histopathological findings showed veins with irregular distension and vascular wall thinning from the mucosa to the submucosal layer. No arteriovenous transition region, a characteristic of arteriovenous malformations, was observed. The lesion was diagnosed as AGD (Fig. 3b). The patient’s postoperative course was satisfactory. She was discharged on the 10th day after surgery, and no re-bleeding has been observed to date at 13 years postoperatively.

## Discussion and conclusion

We experienced one patient with obscure gastrointestinal bleeding due to a jejunal AGD. Lesion localization and stabilization of the patient’s hemodynamics are major challenges in cases of AGDs and other sources of small bowel bleeding that require surgery, especially laparoscopically.

Gastrointestinal AGDs are vascular anomalies caused by acquired vascular malformations that can cause gastrointestinal bleeding [1, 3]. Anatomical factors make small bowel AGDs particularly difficult to diagnose and treat. Progress in DBE technology has meant that surgeons can also choose to treat ongoing hemorrhage by endoscopic hemostasis. However, AGDs cannot always be identified because of their small size and tendency to bleed intermittently [3]. Furthermore, approximately 42.9% of patients experience re-bleeding after endoscopic hemostasis; thus, the most effective approach for stopping bleeding is surgical intervention [4]. The goal of surgery is to remove the bowel segment containing the lesion, but definitively localizing the AGD can be problematic [1, 5–7]. Good localizability is a good indication for laparoscopic surgery, which has spread remarkably in Japan; however, the anatomy of the small intestine makes detection of jejunal lesions difficult. Furthermore, AGDs are small lesions, with a range confined between the mucosal surface and the lamina propria, making it impossible for surgeons to identify them from the serosal side [3, 5, 7, 8].

Many techniques to localize bleeding in the small intestine, including AGDs, have been reported in the literature, including preoperative DBE, angiography, computed tomography-guided dye injection, gastrointestinal bleeding scintigraphy, intraoperative transillumination, intraoperative endoscopy, and segmental clamping [1, 4–6, 9–15]. Ink tattoos injected during DBE or microcoils placed during angiography allow surgeons to identify the lesion site during operations; these landmarks are similarly effective during laparoscopic surgeries [1, 5–7, 9–11]. Transarterial embolization is a reliable approach that allows surgeons to locate bleeding as well as stabilize hemodynamics; however, patients should be operated on quickly thereafter to prevent re-bleeding (if temporary embolic materials were used) or intestinal necrosis. Re-bleeding reportedly occurs in 30–33.3% of patients after arterial embolization for small bowel bleeding [16]. Bleeding scintigraphy can visualize even minor and intermittent hemorrhage; however, its poor ability to localize lesions during surgical operations means that surgeons should utilize a different method [13, 14]. Transillumination is simple to perform but has poor ability in identifying small lesions such as AGDs. Intraoperative examination via endoscopy is time-intensive, which makes it ill-suited to emergency surgeries where the patient is hemodynamically unstable. Segmental clamping is simple and useful for emergency operations with massive bleeding, but tends to result in resection of a greater length of bowel than necessary.

In this patient, the presence of multiple ink tattoos in the small intestine from past DBEs made it impossible to accurately localize the lesion on the basis of these markings alone. The patient exhibited persistent bleeding and unstable hemodynamics as well as signs of extravasation on abdominal angiography. This led us to prioritize stabilizing her hemodynamics, choosing selective arterial embolization to achieve this. Fearing re-bleeding or necrosis of the bowel tissue after embolization, we quickly moved to emergency surgery. Only one of the ink tattoos matched all the landmarks left by the agents administered during the embolization procedure: a cyanoacrylate mass and the catheter tip discovered through palpation, blue dye observed in the adjacent intestinal wall due to indigo carmine, and an intense Lipiodol opacity visualized by fluoroscopy. These signs allowed us to localize the lesion with very high accuracy during the operation. Moreover, this multi-pronged approach was responsible for our swift and accurate localization of the lesion despite the limited operative field available during laparoscopic surgery, and our ability to minimally invasively resect the shortest length of intestine possible (Table 1).

**Table 1:** The methods used for localization of the small intestinal angiodysplasia

Methods	Approach	Identification	Accuracy
Ink tattoo	DBE	Optical	Matched one of the ink tattoos
Catheter tip	Angiography	Palpation	Showed the rough location
Embolization procedure			
Cyanoacrylate	Angiography	Palpation	High accuracy
Indigo carmine dye	Angiography	Optical	High accuracy
Lipiodol	Angiography	Fluoroscopy	High accuracy

*DBE* double-balloon enteroscopy

In conclusion, this case report covered our experience with one patient with gastrointestinal bleeding due to a jejunal AGD. Selective arterial embolization with indigo carmine dye to treat small bowel bleeding preoperatively not only makes the surgery safer by stabilizing the patient’s hemodynamics, but is also very useful for intraoperatively localizing the lesion. Moreover, the high confidence and accuracy of the localization step was largely due to our use of a combination of several embolic agents and dyes during selective arterial embolization.

## Data Availability

Data sharing is not applicable to this article, as no datasets were generated or analyzed during the current study.

## Abbreviations

AGD: Angiodysplasia
DBE: Double-balloon enteroscopy
